# Can You Activate Me? From Robots to Human Brain

**DOI:** 10.3389/frobt.2021.633514

**Published:** 2021-02-19

**Authors:** F. Manzi, C. Di Dio, D. Di Lernia, D. Rossignoli, M. A. Maggioni, D. Massaro, A. Marchetti, G. Riva

**Affiliations:** ^1^ Research Unit on Theory of Mind, Department of Psychology, Università Cattolica del Sacro Cuore, Milan, Italy; ^2^ Humane Technology Lab, Università Cattolica del Sacro Cuore, Milan, Italy; ^3^ Department of Psychology, Università Cattolica del Sacro Cuore, Milan, Italy; ^4^ DISEIS, Department of International Economics, Institutions and Development, Universitá Cattolica del Sacro Cuore, Milan, Italy; ^5^ CSCC, Cognitive Science and Communication research Center, Universitá Cattolica del Sacro Cuore, Milan, Italy; ^6^ Applied Technology for NeuroPsychology Laboratory, Istituto Auxologico Italiano, Milan, Italy

**Keywords:** human-robot interaction, social brain, long-term interaction, fMRI, EEG

**FIGURE 1:**
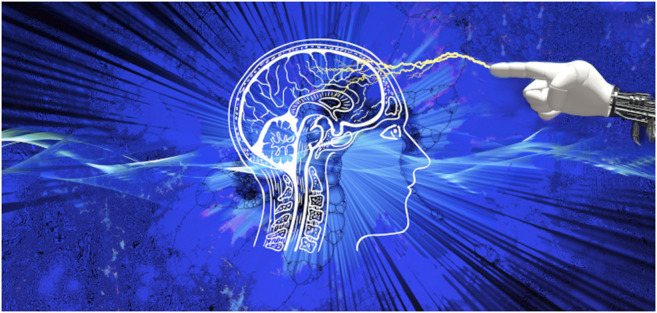
Robots can activate the human brain.

The effectiveness of social robots has been widely recognized in different contexts of humans’ daily life, but still little is known about the brain areas activated by observing or interacting with a robot. Research combining neuroscience, cognitive science and robotics can provide new insights into both the functioning of our brain and the implementation of robots. Behavioural studies on social robots have shown that the social perception of robots is influenced by at least two factors: physical appearance and behavior (Marchetti et al., 2018). How can neuroscience explain such findings? To date, studies have been conducted through the use of both EEG and fMRI techniques to investigate the brain areas involved in human-robot interaction. These studies have mainly addressed brain activations in response to paradigms involving either action performance or charged of an emotional component (Figure 1).

A first set of studies analysed the effect of different types of robots varying in their level of physical anthropomorphism on the activation of the Mirror Neuron Mechanism (MNM). The neuronal activities examined through fMRI indicated that the activation of medial premotor cortex (MPFC) increased linearly over the degree of human-likeness of the robots, from the most mechanical to android ones (Krach et al., 2008). Electroencephalography (EEG) data associated with the mu wave–related to the MNM–showed a modulation of the mu rhythm as a function of the robotic agent resemblance to the human (Urgen et al., 2013; Matsuda et al., 2016). Furthermore, the fMRI findings on MNM indicated that the premotor cortex is similarly activated when actions are performed by different types of robots (more mechanical or android) (Saygin et al., 2012).

These evidences support the hypothesis that the premotor cortex is “automatically” triggered in response to both simple and complex goal-directed and intentional actions, revealing a sensitivity to both the living and non-living ontological status of the agent (Gazzola et al., 2007; Saygin et al., 2012). Activation of the premotor cortex was also found in response to a human or robotic face expressing emotions (Chaminade et al., 2010). Several studies in humans have found that the premotor cortex is involved in the process of emotion recognition by encoding the motor pattern, (i.e. facial expression) that characterizes a given emotional state. The visuo-motor information processed in premotor cortex is translated into affective information by means of the insula that acts as a relay station between the cortical and subcortical areas, such as the amygdala, involved in processing emotional stimuli, (e.g. Carr et al., 2003; Wicker et al., 2003; Iacoboni, 2009). Likewise, the parieto-prefrontal network characterizing the MNM has been found to be particularly sensitive to biological movement, (e.g. Dayan et al., 2007; Casile et al., 2009; Di Dio et al., 2013). Accordingly, it was demonstrated that observing a motor or emotional behaviour performed by a human-like robotic agent, resembling the human kinematics, may be sufficient to activate MNM (Gazzola et al., 2007; Chaminade et al., 2010). Additionally, investigating the vitality forms of movement, which characterize the style of an action, (e.g. rude *vs*. gentle) (Stern, 1985, Stern, 2010), it was shown that, besides the activation of the MNM, vitality forms activate also the dorso-central insular cortex (Di Dio et al., 2013; Di Cesare et al., 2016), which represents the relay through which information about the action style, (i.e. action kinematics) processed in the parietal MNM is invested with an affective quality. Most importantly, very recent neuroscientist evidence has shown that the same brain areas whose activation is stimulated by human vitality forms can be also evoked by robots’ actions performed by simulating human kinematics (Di Cesare et al., 2020), thus conveying information about the robot’s “emotion state”.

However, the activation of other brain areas besides the MNM, such as ventral visual areas, may be required to accommodate the robot’s inconsistent kinematics associated with simple *vs*. complex goal-directed actions (Gazzola et al., 2007). Similarly, fMRI data showed a greater activation of posterior occipital and temporal visual cortices in response to facial expression of robot emotions compared to human emotions, reflecting a further level of processing in response to the unfamiliar stimulus, (i.e. the face of the robot) (Chaminade et al., 2010; Jung et al., 2016). Additionally, the increase in frontal theta activity–associated with the recovery from long-term memory–measured through EEG is greater for a mechanical robot than a human or android (Urgen et al., 2013), highlighting once more the involvement of a compensation process for the analysis of robot stimuli. More specifically, this finding indicates that a lower level of physical robot anthropomorphism requires more resources from memory systems to bridge the semantic gap between the agent and its action (Urgen et al., 2013).

People's sense of affiliation with a robot during interactions is at least partially explained by the emotional responses to the robot's behaviour. Still few studies have analysed the brain activation in response to the emotions expressed by robots. EEG data suggest that people can recognize the bodily emotions expressed by a robot, including joy and sadness, although not all the expressed emotions elicit a significant brain response in the viewer (Guo et al., 2019). Additionally, also fMRI data indicate that emotional expressions, (i.e. joy, anger and disgust) are perceived as more emotional when expressed by a human face than by a robot (Chaminade et al., 2010). As argued above, these differences could be explained by a non-perfect alignment between the robot and human kinematics expressing the emotional quality of movement.

Additional studies had investigated neural activation patterns related to emotional reactions when people observe a robot or a human into a violent situation. The fMRI data showed no differences in activation patterns in areas of emotional resonance when a violent action was experienced by a human or robot (Rosenthal-von der Pütten et al., 2014).

Suzuki et al., (2015) found a similar brain response measured through EEG when observing images showing either a finger of a robotic hand or a human hand getting cut with a scissor. In particular, the authors found an increased neural response in the ascending phase, (i.e. 350–500 ms after stimulus onset) of the P3 component at the frontal-central electrodes by painful human stimuli but not painful robot stimuli, although the difference was only marginal; in contrast, no differences were found for empathy directed toward humans and robots in the descending phase of P3, (i.e. 500–650 ms after stimulus onset). Based on these results, the authors suggest that humanity of the observed agent (human *vs*. robot) partially modulates the top-down controlled processes of empathy for pain (Suzuki et al., 2015), possibly also due to a greater difficulty in taking the robot’s perspective compared to the human one (Suzuki et al., 2015). In this context, it is important to underline that these pioneering studies on empathy are quite heterogenous with respect to both the techniques adopted, and the stimuli used, which vary greatly both in terms of the type of robotic agent and experimental paradigm.

To sum up, our brain systems respond in an “embodied” fashion to the observation of experimental conditions involving the actions of a robot with biological or semi-biological dynamics. However, we suggest that this effect is only transitory or anyway limited to experimental settings. Our consideration is supported by the results by Cross and colleagues (Cross et al., 2019) indicating that a period of real interaction with a social robot can disambiguate its ontological status, thus repositioning the robot in the “non-living category”. This may be plausibly explained by the activation of top-down cognitive mechanisms that regulate the activity of our brain and that highlight the emerge of differences between the brain response to the human *vs*. robot stimuli. In other words, the automatic activation of embodied mechanisms mediated by the MNM when we observe a robot performing actions or experiencing particular human-like emotional states, (e.g. violence or pain) are facilitated in a first “encounter” with the robot, also given our natural tendency to anthropomorphize many different entities. Prior experience with the robot’s actual physical and psychological limits, on the other hand, provides us with a contextual frame of reference whereby top-down processes would modulate or inhibit the response of automatic mechanisms (Paetzel et al., 2020; Rossi et al., 2020). Concluding, although further studies are necessary, we can state that the level of physical anthropomorphism, the type and kinematics of the actions performed by robots jointly activate the social brain areas, consequently increasing the perception of robots as social partners. The use of additional techniques such as Virtual Reality could also prove effective in this respect (Riva et al., 2018; Riva et al., 2019)
